# Adherence and sustained virologic response among vulnerable people initiating an hepatitis C treatment at a nurse-led clinic: A non-experimental prospective cohort study based on clinical records

**DOI:** 10.1016/j.ijnsa.2021.100029

**Published:** 2021-05-26

**Authors:** Myriam Gagné, Isabelle Têtu, Jean-Pierre Grégoire, Jocelyne Moisan

**Affiliations:** a Unity Health Toronto (St. Michael's Hospital), fully affiliated with the University of Toronto, 30, Bond St. (Room Donnelly 6-099), Toronto M5B 1W8, ON, Canada; b Clinique de solidarité SABSA, Québec City, QC, Canada; c Population Health and Optimal Health Practices Research Unit, CHU de Québec–Université Laval Research Center, Québec City, QC, Canada; d Faculty of Pharmacy, Université Laval, Québec City, QC, Canada

**Keywords:** Interprofessional relations, Medication adherence, Nurse-led clinic, Street drugs, Substance-related disorders, Sustained virologic response

## Abstract

**Background:**

There is a need to develop specific care route for vulnerable people with hepatitis C virus.

**Objective:**

At a nurse-led clinic, we aimed to: (1) report the prevalence of patients initiating an hepatitis C treatment who (a) achieved sustained virologic response and (b) were adherent to their treatment; and (2) explore factors associated with adherence and sustained virologic response.

**Design:**

A clinical record-based prospective cohort study.

**Setting:**

A community-based nurse-led clinic coordinating outreach services for people with hepatitis C virus in Québec City, Québec, Canada.

**Population:**

All patients initiating an hepatitis C treatment at the nurse-led clinic from January 1, 2012 to December 31, 2017 (end of data collection).

**Methods:**

Patients were followed from the day they initiated their hepatitis C treatment, up to 24 weeks after the end of the treatment. Sustained virologic response was achieved if hepatitis C virus ribonucleic acid (RNA) was undetectable or below the lower limit of quantification at week 12 or later. Patients who reported hepatitis C treatment completion without missing any doses were considered adherent. Factors associated with adherence and sustained virologic response were identified using adjusted prevalence ratios.

**Results:**

A total of 171 patients infected with hepatitis C virus (women: *n* = 51, 30%; criminal record: *n* = 102, 60%; substance addiction: *n* = 99, 58%) initiated an hepatitis C treatment at the nurse-led clinic. Overall, 126/171 (74%) patients were adherent. Patients using illicit drugs were less likely to be adherent (adjusted prevalence ratio 0.77, 95% confidence interval 0.67–0.89). Among 156/171 (91%) patients with an hepatitis C virus RNA test post-treatment, 96% (*n* = 149) achieved sustained virologic response. Patients who were less likely to achieve sustained virologic response were those who were non-adherent (adjusted prevalence ratio 0.81, 95% confidence interval 0.68–0.98) or who had a criminal record (adjusted prevalence ratio 0.87, 95% confidence interval 0.79–0.97).

**Conclusions:**

A nurse-led clinic could fill an important gap in hepatitis C health services targeted at vulnerable people in a community setting, to drive adherence and achievement of sustained virologic response.

## 1 Introduction

Hepatitis C virus triggers a global pandemic, with 71 million people infected worldwide (The Polaris Observatory HCV Collaborators, 2017). About 75–85% of patients infected with hepatitis C virus will progress to chronic infection (Chen and Morgan, 2006). Over the years, chronic hepatitis C virus infection will slowly and progressively damage the liver, placing people at an increased risk of liver cirrhosis, hepatocellular carcinoma, liver failure, and deaths from hepatitis C (Axley et al., 2018; de Oliveria Andrade et al., 2009; Stanaway et al., 2016).

Evidence shows that anti-hepatitis C virus treatment can cure hepatitis C virus infection in a majority of patients in 8–12 weeks (American Association for the Study of Liver Diseases, Infectious Diseases Society of America, 2019; Miller et al., 2014; Shah et al., 2018). Hence, the primary goal of chronic hepatitis C treatment is to eradicate the virus, termed as sustained virologic response, i.e. absence of viremia 12 weeks after the end of treatment (Jacobson et al., 2012). Sustained virologic response is associated with a 75–90% reduced risk of hepatocellular carcinoma, a 80–97% decreased risk of liver-related mortality, and a 70–90% lower risk of overall mortality (Smith-Palmer et al., 2015).

Antiviral therapy should be considered in all patients with chronic hepatitis C (Backus et al., 2018). Given the improved efficacy, tolerability, and safety of interferon-­free over interferon-based regimens with ribavirin (Feld et al., 2015, McHutchison et al., 2009, Suwanthawornkul et al., 2015, Zeuzem et al., 2018), interferon­free regimens are now recommended (Shah et al., 2018). Several direct-acting antiviral agent regimens, with or without ribavirin, have been approved in Canada and elsewhere (American Association for the Study of Liver Diseases, Infectious Diseases Society of America, 2019; Miller et al., 2014; Shah et al., 2018). Selection of treatment regimens is dependent on various factors, including: previous hepatitis C treatment, liver disease stage, specific comorbidities (e.g. chronic kidney disease, decompensated cirrhosis, post­liver transplantation, hepatitis B coinfection); hepatitis C virus genotype, viral load, and resistance testing (to determine whether adding ribavirin should be considered); and factors accelerating disease progression (Shah et al., 2018). Number of pills per day and duration of treatment should also be considered when selecting treatment regimens (Shah et al., 2018).

In high-income countries, up to 90% of vulnerable people (e.g. those experiencing imprisonment or substance use disorders) are infected with hepatitis C virus (Aldridge et al., 2018). Vulnerable people are commonly excluded from mainstream society as they experience several barriers to treatment engagement and adherence (Luchenski et al., 2018). As a result, they are more likely to experience poor health outcomes (Lewer et al., 2019) and quality of life (Gentil et al., 2019; Lewer et al., 2019). In patients infected with hepatitis C virus, high-drinking and substance addiction have been reported as factors delaying treatment uptake (Socias et al., 2019). Hence, there is a need to develop specific care route to engage the most vulnerable and excluded populations in their hepatitis C care (Luchenski et al., 2018; Popping et al., 2019). Actual interventions, however, have been insufficient to effectively prevent the transmission of HCV among vulnerable people (Larney et al., 2017; Popping et al., 2019).

A nurse-led model of care could facilitate access to evidence-based hepatitis C treatments among those at high risk of sexually transmitted and blood-borne infections, including vulnerable people (Richmond et al., 2020). In a nurse-led approach to care, nurses deliver holistic patient-centered care and consolidate a network of outreach services for vulnerable people, connecting them with gastroenterologists and infectious disease specialists, as well as with other allied healthcare professionals (e.g. pharmacists, nutritionists, outreach workers). Although a nurse-led model of care has been reported to help achieve sustained virologic response among prisoners (Lloyd et al., 2013; Papaluca et al., 2019; Perrett, 2011), it has provided inconclusive results in a community setting (Lewis et al., 2016). Accordingly, we conducted this study with the objective of calculating the prevalence of patients who achieved sustained virologic response after initiating an hepatitis C treatment at a community-based nurse-led clinic (primary objective). Our secondary objectives included: to assess the prevalence of patients who were adherent to their prescribed hepatitis C treatment; and to explore factors associated with treatment adherence and achievement of sustained virologic response.

## 2 Methods

We herein follow the STROBE and RECORD recommendations for reporting of observational non-experimental studies (Benchimol et al., 2015; Vandenbroucke et al., 2007) (*Supplementary Material 1*).

### 2.1 Study design, setting, and population

We conducted a non-experimental prospective cohort study in which we abstracted medical records of all individuals who initiated an hepatitis C treatment at the *Clinique de solidarité SABSA*, a nurse-led clinic located in Québec City, Québec, Canada, from January 1st, 2012 (date at which hepatitis C treatment started at this nurse-led clinic) to December 31st, 2017 (end of data collection). Co-author IT had full access to the database population used to create the study population. The day of initiation of the hepatitis C treatment at the nurse-led clinic marked patients’ entry into the cohort. Standard follow-up visits at the clinic were scheduled at week 1, 2, and 4 after treatment initiation. Follow-up was completed 24 weeks after the end of treatment. A detailed description of healthcare services provided at the clinic is provided as *Supplementary Material 2*.

The present study was approved by the *CHU de Québec* Research Center–*Université Laval* Institutional Ethics Committee (2017-3618, 2016-2017-26 MP). The Ethics Committee did not require researcher to obtain informed consent from individuals whose data were abstracted for the present study.

### 2.2 Variables and measurements

An array of variables was abstracted from the patients’ records by co-author IT with help of a research assistant (the full list of variables can be found in *Supplementary Material 3*). We abstracted the following information recorded prior to treatment initiation: diagnostic date, hepatitis C virus genotype and infection source. We also abstracted various clinical parameters (e.g. alanine aminotransferase; gamma-glutamyl transpeptidase; and liver fibrosis, categorized either as *absent/mild, moderate, severe*, or *advanced*) (de Ledinghen et al., 2006) recorded at hepatitis C treatment initiation. We abstracted data on comorbid physical and mental health problems, patients’ sociodemographic characteristics (e.g. sex; highest attained level of education; and monthly income), self-reported alcohol consumption, current smoking status, and history of recent illicit drug use. Finally, we abstracted the prescribed hepatitis C treatment and its duration (e.g. 8, 12, 24 weeks).

From clinical records, we assessed whether patients had missed at least one dose and whether they had taken the prescribed hepatitis C treatment until the last prescribed dose. We considered patients who reported treatment completion without missing any doses as *adherent*. Patients for whom information on missing doses or treatment completion was lacking were considered as *non-adherent*.

For the period prior to treatment initiation, the treatment period, and the 12-week period following the end of the treatment, we abstracted data on patients’ health services utilization, including the number of encounters with a nurse, a pharmacist, an outreach worker or a gastroenterologist.

We recorded whether sustained virologic response was achieved 12 weeks after the end of treatment (primary outcome). Sustained virologic response was considered achieved if hepatitis C virus ribonucleic acid (RNA) was undetectable or below the lower limit of quantification at week 12 (Jacobson et al., 2012). Patients for whom no information on sustained virologic response was available at week 12 but who achieved sustained virologic response at week 24 were considered as having achieved sustained virologic response at week 12. Those for whom there was no information on sustained virologic response were deemed not having achieved sustained virologic response.

The clinic's nurse partitioner (co-author IT) was the only research team member to have access to clinical records and, therefore, was also the only one in charge of data abstraction. Once completed, all data abstraction forms were checked for reliability by another researcher. Observed invalid, extreme, missing or out-of-range values were reported to co-author IT in charge of data abstraction for additional check. If needed, errors were corrected.

### 2.3 Statistical analyses

All statistical analyses were performed using SAS (Cary, NC, USA). Patients' characteristics were described using frequency distributions, as well as means and standard deviations. Among patients initiating an hepatitis C treatment at the nurse-led clinic, we calculated the prevalence of patients who achieved sustained virologic response and the prevalence of patients who were adherent to the prescribed hepatitis C treatment. A statistician performed univariate regression analyses of the association between study outcomes and each of the independent variables. Variables statistically significant at the 20% level were grouped and a reduced multivariate model was obtained by removing one by one variables not statistically significant at the 5% level of significance. The final multivariate model was fitted using a working-Poisson regression, allowing us to present adjusted prevalence ratios, with associated 95% confidence intervals. Similar analyses were conducted to estimate the association between treatment adherence and the independent variables. Additional details regarding our statistical analyses are provided as *Supplementary Material* 4.

## 3 Results

### 3.1 Flow of patients initiating an hepatitis C treatment at the nurse-led clinic

**Fig. 1:**
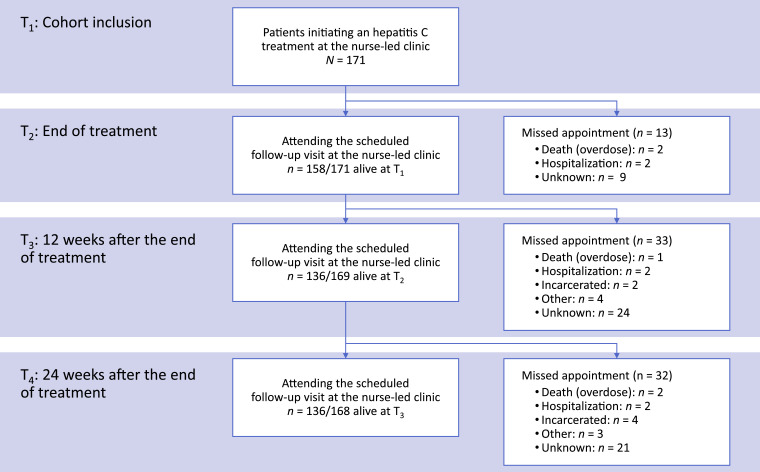
Flowchart of selected patients throughout the study.

From January 1st, 2012 to December 31st, 2017, 171 patients initiated an hepatitis C treatment at the nurse-led clinic. All were included in our analyses. Two patients died from an overdose during the treatment period and 158/169 (93.5%) met a nurse at the end of their treatment. One patient died from an overdose during the 12 weeks that followed. A total of 136/168 patients (81.0%) attended the visit scheduled at week 12 and 136/168 (81.0%) attended the last visit scheduled at week 24. Detailed reasons for missing appointments are provided in Fig. 1.

### 3.2 Characteristics of patients initiating an hepatitis C treatment

Among the 171 patients recruited in this cohort, 120 (70.2%) were male, 120 (70.2%) had less than a college or a university degree, and 95 (55.6%) earned less than US$750 (CA$1,000) per month. About 60% of patients (*n* = 102) had a criminal record (Table 1).

**Table 1:** Sociodemographic characteristics of patients at treatment initiation (*N* = 171).

Characteristics	N	(%)
Sex		
Male	120	(70.2%)
Female	51	(29.8%)
Sexual orientation		
Heterosexual	165	(96.5%)
Other	6	(3.5%)
Age (years)		
22 to 45: quantile 1	45	(26.3%)
46 to 52: quantile 2	47	(27.5%)
53 to 59: quantile 3	39	(22.8%)
60 to 75: quantile 4	40	(23.4%)
Country of birth		
Canada	162	(94.7%)
Other	9	(5.3%)
Marital status		
Single	109	(63.7%)
In relationship	62	(36.3%)
Residential status		
Housed	159	(93.0%)
Homeless (including shelters and transitional housing)	12	(7.0%)
Place of residence		
Québec City, *La Cité-Limoilou* district (where *SABSA* is located)	91	(53.2%)
Québec City, any other district	41	(24.0%)
Outside Québec City, but within the *Capitale-Nationale* administrative region	13	(7.6%)
Outside the *Capitale-Nationale* administrative region	26	(15.2%)
Highest attained level of education		
Elementary school	61	(35.7%)
High school diploma	40	(23.4%)
Diploma of vocational studies	19	(11.1%)
College degree	24	(14.0%)
≥Bachelor's degree	10	(5.9%)
Missing data	17	(9.9%)
Primary occupation		
Work or study	59	(34.5%)
Retired	15	(8.8%)
Unemployed	97	(56.7%)
Monthly income^a^		
From US$375 to US$749	95	(55.6%)
≥US$750	75	(43.9%)
Missing data	1	(0.5%)
Medication insurance coverage		
Public	139	(81.3%)
Private	32	(18.7%)
Had a criminal record		
No	69	(40.4%)
Yes	102	(59.6%)

a Data on monthly income were collected in Canadian dollars: As of February 12, 2020, CA$ 1 = US$ 0.753761.

As shown in Table 2, about half of patients were overweight or obese (*n* = 90, 52.6%). About 80% of individuals had at least one comorbid mental health problem, the most common being substance addiction (*n* = 99, 57.9%). Illicit injection drug use in the past 30 days was self-reported by 37 patients (21.6%), with 25 and 19 patients reporting having injected cocaine and opioid, respectively (Table 3). About 40% of patients (*n* = 68) reported recent use of illicit non-injection drugs such as amphetamine (*n* = 30) and cannabis (*n* = 46).

**Table 2:** Clinical characteristics of patients at treatment initiation (*N* = 171).

Clinical characteristics	N	(%)
**Patients**		
Body mass index (kg/m^2^)		
<18.5: underweight	4	(2.3%)
18.5 to 24.9: normal weight	55	(32.2%)
25 to 29.9: overweight	58	(33.9%)
>30: obese	32	(18.7%)
Missing data	22	(12.9%)
Had any of the following physical health problems:	115	(67.3%)
Comorbid physical health problem:		
Cancer (other than hepatocellular carcinoma)	3	(1.8%)
Hepatocellular carcinoma	2	(1.2%)
Diabetes	26	(15.2%)
Chronic pain	35	(20.5%)
Hepatitis B	9	(5.3%)
Cardiovascular disease	41	(24.0%)
Chronic inflammatory disease	16	(9.4%)
Chronic pulmonary disease	39	(22.8%)
Chronic kidney disease	10	(5.9%)
Thromboembolic disease	2	(1.2%)
Human immunodeficiency virus/acquired immune deficiency syndrome^a^	7	(4.1%)
Had any of the following mental health problems:	135	(79.0%)
Comorbid mental health problem:		
Anxiety	52	(30.4%)
Alcoholism	40	(23.4%)
Bipolarity	6	(3.5%)
Schizophrenia	7	(4.1%)
Substance addiction	99	(57.9%)
Adjustment disorder	2	(1.2%)
Attention deficit disorder	20	(11.7%)
Personality trouble	60	(35.1%)
Active major depressive disorder, first episode	2	(1.2%)
Recurrent major depressive disorder	52	(30.4%)
Other mental health problem	4	(2.3%)
Alanine aminotransferase (U/L)		
≥0 and ≤50^b^	67	(39.2%)
>50	85	(49.7%)
Missing data	19	(11.1%)
Total bilirubin (mmol/L)		
≥0 et ≤21^b^	121	(70.8%)
>21	8	(4.7%)
Missing data	42	(24.6%)
Creatinine (μml/L)		
<55	13	(7.6%)
≥55 et ≤105^b^	121	(70.8%)
>105	10	(5.9%)
Missing data	27	(15.8%)
Gamma-glutamyl transpeptidase (U/L)		
<50^b^	55	(32.2%)
≥50	67	(39.2%)
Missing data	49	(28.7%)
Haemoglobin (g/L)		
<140	66	(38.6%)
≥140 et ≤180^b^	91	(53.2%)
Missing data	14	(8.2%)
Platelet count (10^9^/L)		
<150	48	(28.1%)
≥150 et ≤400^b^	110	(64.3%)
Missing data	13	(7.6%)
Thyroid-stimulating hormone (mUI/L)		
<0,25	1	(0.6%)
≥0,25 et ≤5^b^	123	(71.9%)
>5	5	(2.9%)
Missing data	42	(24.6%)
**Hepatitis C virus**		
Hepatitis C virus genotype		
1a	93	(54.4%)
1b	15	(8.8%)
2	13	(7.6%)
3	43	(25.1%)
4, 5, 6 and 1+3	7	(4.1%)
Hepatitis C virus infection		
Injection drug use	140	(81.9%)
Other mode of transmission^c^	31	(18.1%)
Liver fibrosis		
Absent, mild or severe fibrosis (F3-F0)	74	(43.3%)
Advanced fibrosis (F4) or cirrhosis	77	(45.0%)
Missing data	20	(11.7%)

a These seven people who were co-infected with human immunodeficiency virus had an undetectable viral load.

b Normal values.

c Other modes of transmission include: using intranasal drugs; recipients of blood transfusions; being born to a mother infected with hepatitis C virus; unregulated tattooing and piercing in prisons, etc.

**Table 3:** Recent illicit drug, alcohol, and tobacco use by patients before treatment initiation (*N* = 171).

Substance used in the past 30 days	N	(%)
Illicit injection drug		
None used	134	(78.4%)
Used only one drug	29	(17.0%)
Used ≥2 drugs	8	(4.7%)
Injected:		
Amphetamine	1	(0.6%)
Cocaine	25	(14.6%)
Opioid	19	(11.1%)
Another injection drug	1	(0.6%)
Illicit non-injection drug		
None used	103	(60.2%)
Used only one drug	45	(26.3%)
Used ≥2 drugs	23	(13.5%)
Used:		
Amphetamine	30	(17.5%)
Cocaine or crack	13	(7.6%)
Cannabis	46	(26.9%)
Opioid	5	(2.9%)
Gamma hydroxybutyrate (GHB) drug	1	(0.6%)
Another stimulant	1	(0.6%)
Number of standard alcohol drinks per week^a^		
0	103	(60.2%)
1 to 7	24	(14.0%)
8 to 14	8	(4.7%)
15 to 21	9	(5.3%)
>21	26	(15.2%)
Drink alcohol, but could not recall how many drinks a week	1	(0.6%)
High-risk drinking	37	(21.6%)
Current tobacco smoker	112	(65.5%)

a In women, low-risk drinking is defined as no more than 10 standard drinks a week. In men, low-risk drinking is defined as no more than 3 drinks a day or 15 standard drinks a week. One standard drink is equivalent to one regular beer (340 ml/12 oz, 5% alcohol), one glass of wine (140 ml/5oz, 12% alcohol), one glass of fortified wine (85 ml/3oz, 20% alcohol) and one shot of spirits (45 ml/1.5 oz, 40% alcohol).

### 3.3 Characteristics of hepatitis C treatments

A total of 56 patients (32.7%) have had a prior hepatitis C treatment before entering the cohort. These patients were seeking another treatment either due to prior treatment failure (*n* = 50, 89.2%) or due to hepatitis C virus re-infection (*n* = 6, 10.7%). Patients (*n* = 166, 97.1%) were mostly prescribed the hepatitis C drug treatment by a gastroenterologist. Two-thirds of patients (*n* = 106, 62.0%) were prescribed a direct-acting antiviral without ribavirin (*Supplementary Material 5*). While 133 patients (77.8%) were treated for 8 or 12 weeks, 8 patients (4.7%) had a treatment duration of 48 weeks.

### 3.4 Adherence to the hepatitis C treatment

Due to adverse reactions, physicians prescribed hepatitis C treatment discontinuation for eight patients. A total of 158 patients attended the visit scheduled at the end of the hepatitis C treatment (hence, 6 patients [3.5%] were deemed to be non-adherent due to missing information for this outcome in their record). Among these 158 patients, 93% (*n* = 147) reported having completed the treatment and 126 (85.7%) also reported having taken all prescribed doses. These 126 patients (73.7% of the cohort) were considered as adherent to their prescribed treatment.

### 3.5 Characteristics associated with adherence to the hepatitis C treatment

Results of univariate regression analyses of the association between treatment adherence and each of the independent variables are presented as *Supplementary Material 6*. Multivariate analyses showed that patients reporting a recent use of illicit drugs were less likely to be adherent (recent use *vs.* non-use of illicit drugs: adjusted prevalence ratio 0.77, 95% confidence interval 0.67–0.89). Patients who were prescribed an hepatitis C treatment length of 48 weeks (*vs.* 8 or 12 weeks) were also less likely to be adherent to their hepatitis C treatment (adjusted prevalence ratio 0.29, 95% confidence interval 0.09–0.94).

### 3.6 Health services used during the treatment period

Prior to and during the hepatitis C treatment period, all patients were seen by a nurse (Table 4). Nearly all patients (*n* = 163, 95.3%) met a gastroenterologist prior to initiating the treatment, while less than half of patients (*n* = 71, 44.9%) met a gastroenterologist during the treatment period. During the treatment period, about half of patients attended more visits at the clinic than what was scheduled (43.0%).

**Table 4:** Health services use by the 171 patients before, during, and after treatment.

Health services	From the first visit at the nurse-led clinic to treatment initiation (*N* = 171)		During the course of the hepatitis C treatment (*N* = 158)		During the 12-week period following treatment cessation (*N* = 136)	
	N	(%)	N	(%)	N	(%)
Was hospitalized	10	(5.9%)	8	(5.1%)	2	(1.5%)
Visited the emergency	10	(5.9%)	13	(8.2%)	5	(3.8%)
Consulted a nutritionist	5	(2.9%)	2	(1.3%)	0	(0%)
Consulted a pharmacist	77	(45.0%)	2	(1.3%)	0	(0%)
Consulted an outreach worker	87	(50.9%)	73	(46.2%)	51	(38.6%)
Consulted a nurse	171	(100.0%)	158	(100.0%)	131	(99.2%)
Consulted a general practitioner	33	(19.3%)	37	(23.4%)	24	(18.2%)
Consulted an infectious disease physician	6	(3.5%)	6	(3.8%)	7	(5.3%)
Consulted a gastroenterologist	163	(95.3%)	71	(44.9%)	52	(39.4%)
Consulted another specialist doctor	27	(15.8%)	22	(13.9%)	14	(10.6%)
Received services from a community organization	68	(39.8%)	67	(39.2%)	53	(39.9%)
Mean number (±standard deviation) of visits scheduled at the nurse-led clinic	N/A	N/A	5.8	(± 4.2)	2.9	(± 0.7)
Mean number (±standard deviation) of visits scheduled at the nurse-led clinic that patients attended	N/A	N/A	6.9	(± 4.9)	4.0	(± 2.4)
Difference between the number of visits scheduled and the number of visits that occurred at the nurse-led clinic						
Lower number	N/A	N/A	29	(18.4%)	14	(10.6%)
Equal number	N/A	N/A	61	(38.6%)	53	(40.2%)
Higher number	N/A	N/A	68	(43.0%)	65	(49.2%)

N/A: Not applicable

### 3.7 Sustained virologic response

Among the 171 patients who initiated an hepatitis C treatment, 133 (77.8%) had an hepatitis C virus RNA test 12 weeks after the end of treatment. Of these 133 patients, 95.5% (*n* = 127) achieved sustained virologic response. Twenty-four weeks after the end of treatment, 135 patients had an hepatitis C virus RNA test (including 23 patients who did not have an hepatitis C virus RNA test 12 weeks before). Of these 135 patients, 127 (93.4%) achieved sustained virologic response. Only one person was re-infected with hepatitis C virus due to injection drug use. The remaining 7 patients did not achieve sustained virologic response at week 24 due to treatment failure. A total of 156 patients had an hepatitis C virus RNA test during the study period, of whom 149 (95.5%) achieved sustained virologic response (15 patients were deemed not to have achieved sustained virologic response due to missing data for this outcome in their record). The 149 patients who achieved sustained virologic response correspond to 87.1% of the cohort.

### 3.8 Characteristics associated with achievement of sustained virologic response

Results of univariate regression analyses of the association between sustained virologic response achievement and each of the independent variables are presented as *Online Supplementary Material 7*. In multivariate analyses, patients who had a criminal record were less likely to achieve sustained virologic response (patients who had a criminal record *vs.* not: adjusted prevalence ratio 0.87, 95% confidence interval 0.79–0.97). Patients who were non-adherent to their hepatitis C treatment were also less likely to achieve sustained virologic response (non-adherent *vs.* adherent adjusted prevalence ratio 0.81, 95% confidence interval 0.68–0.98).

## 4 Discussion

### 4.1 Key findings

First, this prospective cohort study shows that a high proportion of vulnerable people initiating an hepatitis C treatment at a community-based nurse-led clinic adhered to their hepatitis C treatment and achieved sustained virologic response. Second, an hepatitis C treatment length of 48 weeks (*vs.* 8 or 12 weeks) and recent use of illicit drugs (*vs.* non-use) were identified as key independent factors associated with a lower likelihood of adherence in our study population. Third, patients with a criminal record and those who were non-adherent to the prescribed hepatitis C treatment were less likely to achieve sustained virologic response.

### 4.2 Explanation of findings

Our results show that more than half of patients initiating an hepatitis C treatment at the nurse-led clinic had a criminal record and were considered as having substance addictions. When provided with nurse-led care in the community, a high proportion of these vulnerable people not only engage in their care by attending all their scheduled follow-up visits, but are also adherent to their treatment and, in turn, achieve sustained virologic response. As such, this study adds to a growing body of evidence from studies conducted in prison settings suggesting that nurse-led clinic can safely and efficiently coordinate interdisciplinary hepatitis C care (Perrett, 2011; Tait et al., 2010), as well as improve treatment uptake (Lloyd et al., 2013) and outcomes in people with hepatitis C (Overton et al., 2019; Papaluca et al., 2019). Our results nevertheless contrast with those of a cluster randomized controlled trial comparing nurse-initiated vs. physician-initiated hepatitis C therapy in a community setting (Lewis et al., 2016). In this trial, where nurses’ role was limited to offering immediate antiviral therapy, uptake and adherence remained poor in vulnerable people and were not different between the two groups (Lewis et al., 2016). By contrast, in the present study, services provided at the nurse-led clinic were both tailored to the specific needs of the vulnerable populations and highly consistent with strategies developed to improve satisfaction with care among vulnerable people (Gentil et al., 2020). For instance, patients could attend all their hepatitis C-related medical appointments in a strategic location, close to where they lived (a key enabler to treatment engagement among vulnerable people) (Wolstenholme et al., 2020). To avoid transport-related problems, outreach workers picked up patients for their appointments and drove them to the clinic. Nurses who acted as case managers ensured continuity of care (Gentil et al., 2020) as well as provision of health and social services that tackled not only the physical and mental illnesses of vulnerable patients, but also their addictions, as others called for (Luchenski et al., 2018). As such, services provided at the community-based nurse-led clinic may have driven its apparent success in terms of uptake, adherence, and sustained virologic response.

Our findings suggest that recent illicit drug use (*vs.* non-use) and hepatitis C treatment duration (48 weeks *vs.* 8 or 12 weeks) are key factors associated with a reduced likelihood of treatment adherence. Treatment non-adherence and having a criminal record were identified as independent factors associated with a lower likelihood of sustained virologic response achievement. These findings are consistent with those from a previous meta-analysis that showed that recent illicit drug use was associated with non-adherence (Hajarizadeh et al., 2018). Treatment adherence has also been reported in the past to be the strongest predictor for sustained virologic response (Vo et al., 2015).

### 4.3 Future directions

Effective treatments are widely available and concerted efforts to eliminate hepatitis C virus as a public health threat have been called for by the World Health Organization (World Health Organization, 2016). In this context, our findings are noteworthy, not only because hepatitis C treatment uptake by vulnerable people is crucial to meet the 2030 hepatitis C virus elimination target (World Health Organization, 2016), but also because studies have shown that people with substance addictions and those with low monthly income are less likely to undertake an hepatitis C treatment than others (Nitulescu et al., 2019; Saeed et al., 2020). Our results suggest that a nurse-led clinic can successfully reach vulnerable populations in a community setting, in order to assist them in being cured from hepatitis C virus. There is now a need to assess the comparative impact of this nurse-led model of care for vulnerable people, compared to usual physician-led care, on adherence, sustained virologic response, but also healthcare costs.

### 4.4 Strengths and limitations

This study has several strengths. All individuals initiating an hepatitis C treatment at the nurse-led clinic during the study period were included. We were able to comprehensively describe patients, hepatitis C virus infections, and hepatitis C treatments received. We were also able to study an array of sociodemographic, clinical, and behavioural characteristics potentially associated with treatment adherence and sustained virologic response achievement.

This study has also limitations. First, data used in this study were originally collected for clinical purpose. Data misclassification may therefore have occurred (with patients potentially underreporting illicit drug use). Second, patients for whom we found in their clinical record no information on treatment completion (3.5% of patients) and on sustained virologic response (8.8% of patients) were considered as non-adherent and as not having achieved virologic response, respectively. Therefore, the proportion of patients who were found to be adherent to their treatment and the proportion of those who achieved sustained virologic response may have been underestimated. Finally, in the absence of a comparison group, we were not able to assess the effectiveness of this nurse-led model of care compared to standard physician-led care. The results of this present study send a potential efficacy signal.

## 5 Conclusions

Our results suggest that a nurse-led clinic offering outreach services for people infected with hepatitis C virus could fill an important gap in hepatitis C health services delivered in a community setting. We believe these results can be generalized to patients who initiate an hepatitis C treatment in clinics that serve vulnerable patient populations similar to those enrolled in this cohort.

## Tweetable abstract

High adherence and sustained virologic response in vulnerable people initiating an #Hepc treatment at a community-based #NPsLead clinic.

**Contribution of the paper**

**What is already known about the topic**

Hepatitis C virus triggers a global pandemic. Effective treatments, including direct-acting antivirals, are available and can cure hepatitis C virus infection in most patients.

In high-income countries, up to 90% of vulnerable people are infected with hepatitis C virus. Adherence to hepatitis C treatment is suboptimal in this population.

There is a need to develop specific care route to engage the most vulnerable populations in their hepatitis C care. Actual interventions, however, have been insufficient to effectively prevent the transmission of hepatitis C virus among vulnerable people in a community setting.

**What this paper adds**

This prospective cohort study shows that a community-based nurse-led clinic engages vulnerable people in their care. Patients attend their follow-up visits, adhere to their hepatitis C treatment, and achieve sustained virologic response.

This study identifies hepatitis C treatment length of 48 weeks (*vs.* 8 or 12 weeks) and recent use of illicit drugs (*vs.* non-use) as independent factors associated with a lower likelihood of adherence.

This study suggests that patients with a criminal record and those who are non-adherent to the prescribed hepatitis C treatment might be less likely to achieve sustained virologic response.

**List of abbreviations**

RNA: Ribonucleic acid

## Authors' contributions

All authors contributed to the study design. MG wrote the initial draft and [IT, JPG, JM revised it for important intellectual content. All authors have given final approval of the version to be published and agree to be accountable for all aspects of the work.

## Declaration of competing interest

The authors declare that they have no known competing financial interests or personal relationships that could have appeared to influence the work reported in this paper.

## Appendix Supplementary materials

Image, application 1

Image, application 2

Image, application 3

Image, application 4

Image, application 5

Image, application 6
