# Supplementary material for: Adherence and sustained virologic response among vulnerable people initiating an hepatitis C treatment at a nurse-led clinic: A non-experimental prospective cohort study based on clinical records
Source: Int J Nurs Stud Adv. 2021 May 26;3:100029. doi: 10.1016/j.ijnsa.2021.100029 (PMC11080331; doi:10.1016/j.ijnsa.2021.100029)
Supplement: Supplementary file 1 [file mmc1.docx]

## Additional File 2

#### Healthcare Services Provided at the Nurse-Led Clinic

##### Initial diagnosis and patient referral

Individuals infected with hepatitis C virus are referred to the clinic either by a gastroenterologist, an infectious disease specialist, an outreach worker, or by any other healthcare professionals from the public health services network. Patients can also come in for screening services or because they know or suspect that they are infected with hepatitis C virus. In such cases, nurses schedule an appointment with a physician for hepatitis C diagnosis.

##### First visit and hepatitis C treatment prescription

During the first visit, nurses review patients’ medical history and perform physical and mental examinations. They collect venous blood for detecting hepatitis C virus ribonucleic acid (RNA)load and genotyping. They schedule patients for transient elastography (FibroScan; Echosens, Paris, France). Nurses interview patients about risky behaviours (e.g.: smoking habits, alcohol consumption, recent use of illicit drugs such as cocaine, opioid, or cannabis) and use of prescribed medications. They provide them with additional healthcare services, including vaccination against hepatitis A and B, influenza, and tetanus.

When necessary, nurses refer patients to outreach workers for assistance in finding accommodation or food, undergoing therapy to overcome addictions, or getting a card for health services coverage by the Public Health Insurance Board.

##### Prescription for hepatitis C treatment

Upon receipt of blood test results, physicians collaborating with the nurse-led clinic prescribe the hepatitis C treatment. Nurses forward prescriptions to collaborating pharmacists, who assess therapeutic appropriateness, perform medication reviews, and take steps to prevent drug-related problems, including recommending physicians to adapt prescriptions to avoid drug-drug interactions. When necessary, nurses communicate with physicians in order to modify the prescription.

##### Approval for hepatitis C medications

Nurses seek approval for reimbursement of HCV medications from private insurers or from the Public Health Insurance Board, depending on whether patients are covered by private insurance or by the public drug plan. Authorizations are usually obtained within three to six weeks.

##### Treatment initiation and follow-up

Nurses meet all patients at treatment initiation for education and counselling. During this visit, venous blood is collected for assessment of different non-invasive biochemical markers for the diagnosis of cirrhosis, including: creatinine, alanine aminotransferase (ALT); aspartate aminotransferase (AST); gamma-glutamyl transpeptidase (GGT); total bilirubin; complete blood count; prothrombin time; and albumin. Nurses and patients reach agreement upon the date of the beginning of the HCV treatment. Prescriptions are then faxed to each patient’s community pharmacy. Nurse practitioners give a call to pharmacists to inform them about their contact information.

During the treatment period, standard follow-up visits occur at week 1, 2, and 4 after treatment initiation to foster patient engagement and optimize adherence. Additional appointments can be scheduled during the treatment period. The number of additional appointments and the time interval between them vary according to physicians’ requirements and patients’ needs. When necessary, outreach workers meet patients at home and assist them in attending their follow-up visits, e.g. by picking them up and driving them to the clinic. At each follow-up visit, venous blood is collected by nurses. Treatment efficacy, safety, and use are assessed at each visit. Treatment compliance is measured by nurses by asking patients: *“Since your last appointment, did you forget to take any dose of your HCV medications?”* Pharmacists also communicate with nurses when patients do not pick up HCV medications. In this case, nurses or outreach workers communicate with patients to discuss and address barriers to HCV treatment adherence.

At the end of treatment, nurses meet patients. During this meeting, nurses ask patients whether they took the treatment until the last dose prescribed, collect venous blood and educate patients regarding risky behaviours and risks of relapse of HCV.

After this visit, outreach workers continue to assist patients for other physical and mental health problems. To determine whether patients achieve sustained virologic response, hepatitis C virus RNA load is assessed 12 and 24 weeks after the end of the treatment (Jacobson et al., 2012).

### References

Jacobson, I.M., Poordad, F., Brown, R.S., Jr., Kwo, P.Y., Reddy, K.R., Schiff, E., 2012. Standardization of terminology of virological response in the treatment of chronic hepatitis C: panel recommendations. J Viral Hepat 19 (4), 236-243.
