# Supplementary material for: Adherence and sustained virologic response among vulnerable people initiating an hepatitis C treatment at a nurse-led clinic: A non-experimental prospective cohort study based on clinical records
Source: Int J Nurs Stud Adv. 2021 May 26;3:100029. doi: 10.1016/j.ijnsa.2021.100029 (PMC11080331; doi:10.1016/j.ijnsa.2021.100029)
Supplement: Supplementary file 2 [file mmc2.docx]

## Additional File 3

#### Methods: Additional Details Regarding Variables and Measurements

Considering our study objectives, we abstracted the following variables from each participant’s clinical record.

##### Characteristics of HCV and participants

From clinical records, we abstracted the following data in regard to hepatitis C recorded during the period beginning the day of the first visit to the nurse-led clinic and ending on the day before treatment initiation: diagnostic date; hepatitis C virus genotype; and infection source.

We also abstracted recorded data at the beginning of the hepatitis C treatment on the following clinical parameters: alanine aminotransferase (IU/L); aspartate aminotransferase (IU/L); gamma-glutamyl transpeptidase (IU/L); total bilirubin (μmol/L); platelet count (G/L); prothrombin time (%); albumin (g/L); and liver fibrosis (categorized either as *advanced* [F4], *severe* [F3], *moderate* [F2], *mild/absent* [F0-1]) (de Ledinghen et al., 2006). Co-infection with hepatitis C and human immunodeficiency virus was abstracted. In patients who were co-infected by hepatitis C and human immunodeficiency virus, we recorded the human immunodeficiency virus viral load. We additionally abstracted data on: weight and height (for body mass index calculation); comorbid physical health problems such as: cancer (including hepatocellular carcinoma), diabetes, chronic pain, hepatitis B, cardiovascular disease, chronic inflammatory disease, chronic pulmonary disease, chronic kidney disease, and thromboembolic disease; comorbid mental health problems, including: anxiety, alcoholism, bipolarity, schizophrenia, substance addiction, adjustment disorder, attention deficit disorder, personality trouble, and active major depressive disorder (first and recurrent episodes). We also abstracted data related to the following patients’ sociodemographic characteristics: country of birth (Canada or elsewhere); date of birth (this enabled us to calculate age at *treatment initiation*); sex; highest attained level of education; marital status; sexual orientation; and monthly income. Finally, we abstracted the following behavioural variables regarding illicit drug: self-reported history of injecting and non-injecting drug use in the 30-day prior to treatment initiation; and if so, self-reported drugs used (e.g. amphetamine; cocaine; crack; opioid; cannabis). Regarding alcohol consumption, we abstracted the self-reported weekly number of standard alcohol drinks and dichotomized it as low- and high-risk drinking (the former defined as no more than 10 standard drinks a week for women and no more than 15 drinks a week for men). We also abstracted whether patients were current smoker.

##### Prescribed treatment

For each patient, we abstracted the following data currently recorded on the first day of the hepatitis C treatment: (1) the international non-proprietary drug (i.e. generic) name and (2) the length of the prescribed HCV treatment. We also abstracted whether patients were vaccinated against hepatitis A and B, as well as all other medications concomitantly prescribed or used by patients.

##### Adherence to hepatitis C treatment

Adherence to hepatitis C treatment was assessed based on patients’ records. At the end of treatment patients are asked by nurses whether they have taken the prescribed treatment until the last prescribed dose. Patients for whom it was mentioned in their file that they had reported hepatitis C treatment completion without missing any doses were defined as *adherent*. Patients for whom information on missing dose or treatment completion was lacking were considered as *non-adherent*.

##### Adverse reaction

From clinical records, during the treatment period, we abstracted data on patients’ self-reported adverse reactions.

##### Health service utilization

From clinical records, for the period between the first visit and the day before treatment initiation, during the treatment period, and during the 12 weeks following the end of treatment, we abstracted all health services used by patients. We abstracted whether patients had been hospitalized or had an emergency department visit. In addition, the number of encounters with nutritionists, pharmacists, outreach workers, nurses, general practitioners, infectious disease specialists, gastroenterologists and any other physician specialist were abstracted. We also collected data on whether patients had received services from a community organization. Finally, we abstracted the number of visits that were scheduled and the number of visits that actually occurred.

##### Sustained virologic response

From clinical records, we assessed whether sustained virologic response was achieved 12 weeks after the end of treatment (primary outcome). Patients for whom no information on sustained virologic response was found in their record at 12 weeks but who achieved sustained virologic response at 24 weeks were considered as having achieved sustained virologic response at 12 weeks, because no individuals undertook a new HCV treatment during this period. Those for whom there was no information at all on sustained virologic response were deemed not having achieved sustained virologic response.

## References

de Ledinghen, V., Douvin, C., Kettaneh, A., Ziol, M., Roulot, D., Marcellin, P., Dhumeaux, D., Beaugrand, M., 2006. Diagnosis of hepatic fibrosis and cirrhosis by transient elastography in HIV/hepatitis C virus-coinfected patients. J Acquir Immune Defic Syndr 41 (2), 175-179.
