# Supplementary material for: Adherence and sustained virologic response among vulnerable people initiating an hepatitis C treatment at a nurse-led clinic: A non-experimental prospective cohort study based on clinical records
Source: Int J Nurs Stud Adv. 2021 May 26;3:100029. doi: 10.1016/j.ijnsa.2021.100029 (PMC11080331; doi:10.1016/j.ijnsa.2021.100029)
Supplement: Supplementary file 3 [file mmc3.docx]

## Additional File 4

#### Methods: Additional Details Regarding Statistical Analyses

All statistical analyses were performed using SAS (Cary, NC, USA). Patient characteristics were described using frequency distributions, as well as means and standard deviations. We calculated the prevalence of patients who achieved sustained virologic response and the prevalence of individuals who were adherent to the prescribed hepatitis C treatment. We performed univariate regression analyses of the association between sustained virologic response achievement and each of the independent variables. In these analyses, sustained virologic response achievement was calculated using an intention-to-treat approach (i.e.: patients with a missing outcome value were deemed as not having achieved sustained virologic response). Variables statistically significant at the 20% level were then grouped into five blocks: (1) personal characteristics (e.g.: age and sex), (2) socioeconomic status (e.g. education and income), (3) health status (e.g. comorbid physical or mental problem), (4) health behavior (e.g. illicit drug use) and (5) non-adherence to hepatitis C treatment. A reduced multivariate model was obtained for each block by removing one by one variables not statistically significant at the 20% level. Variables remaining in each of the five reduced models were put together and a final multivariate model was obtained by further removing variables using a 5% level of significance. Given that some variables showed 100% sustained virologic response achievement in one of their categories, there was a need to conduct exact tests. For these analyses, at first, we had planned to calculate prevalence ratios using working-Poisson regressions (i.e. generalized linear models with log link, Poisson distribution and robust variance). However, since in the SAS software working-Poisson regression does not allow to conduct exact tests, instead we had to use logistic regression to build the multivariate model. For the final multivariate model, given the retained variables, exact tests were not needed (i.e.: there was not 100% sustained virologic response achievement in one of their categories). The final multivariate model was therefore re-fitted using working-Poisson regression, allowing to present prevalence ratios for the final multivariate model. Similar analyses were conducted to estimate the association between patient adherence and the independent variables.
