# Supplementary material for: Adherence and sustained virologic response among vulnerable people initiating an hepatitis C treatment at a nurse-led clinic: A non-experimental prospective cohort study based on clinical records
Source: Int J Nurs Stud Adv. 2021 May 26;3:100029. doi: 10.1016/j.ijnsa.2021.100029 (PMC11080331; doi:10.1016/j.ijnsa.2021.100029)
Supplement: Supplementary file 4 [file mmc4.docx]

# Supplementary Material 5

## e-Table 1. Hepatitis C Treatment Regimens Prescribed to the 171 Patients

| International non-proprietary (generic) name |  | **Duration of treatment  (weeks)** |  | **N** |  | **(%)** |
| --- | --- | --- | --- | --- | --- | --- |
| **Direct-acting antiviral without ribavirin** |  |  |  | 106 |  | (62.0%) |
| elbasvir/grazoprevir |  | 12 |  | 19 |  | (11.1%) |
| ledispavir/sofosbuvir |  | 8 |  | 9 |  | (5.3%) |
| ledispavir/sofosbuvir |  | 12 |  | 35 |  | (20.5%) |
| ledispavir/sofosbuvir |  | 24 |  | 2 |  | (1.2%) |
| ombitasvir/paritaprevir/ritonavir |  | 12 |  | 1 |  | (0.6%) |
| paritaprevir/ritonavir/ombitasvir + dasabuvir |  | 12 |  | 3 |  | (1.8%) |
| paritaprevir/ritonavir/ombitasvir + dasabuvir + ledispavir/sofosbuvir |  | 12 |  | 1 |  | (0.6%) |
| paritaprevir/ritonavir/ombitasvir + dasabuvir + simeprevir |  | 12 |  | 1 |  | (0.6%) |
| sofosbuvir/velpatasvir |  | 12 |  | 33 |  | (19.3%) |
| sofosbuvir/velpatasvir/voxilarevir |  | 12 |  | 2 |  | (1.2%) |
| **Direct-acting antiviral with ribavirin** |  |  |  | 37 |  | (21.6%) |
| daclastavir+sofosbuvir+ribavirin |  | 12 |  | 1 |  | (0.6%) |
| daclastavir+sofosbuvir+ribavirin |  | 24 |  | 1 |  | (0.6%) |
| ledispavir/sofosbuvir + ribavirin |  | 12 |  | 6 |  | (3.5%) |
| ledispavir/sofosbuvir + ribavirin + sofosbuvir |  | 24 ^a^ |  | 1 |  | (0.6%) |
| paritaprevir/ritonavir/ombitasvir + dasabuvir + ribavirin |  | 12 |  | 8 |  | (4.7%) |
| simeprevir + sofosbuvir + ribavirin |  | 12 |  | 1 |  | (0.6%) |
| Sofosbuvir + ribavirin |  | 12 |  | 8 |  | (4.7%) |
| Sofosbuvir + ribavirin |  | 24 |  | 10 |  | (5.8%) |
| sofosbuvir/velpatasvir + ribavirin |  | 12 |  | 1 |  | (0.6%) |
| **Interferon-based hepatitis C treatment with ribavirin** |  |  |  | 28 |  | (14.4%) |
| interferon pegyle alfa 2a/ribavirin |  | 24 |  | 7 |  | (4.1%) |
| interferon pegyle alfa 2a /ribavirin |  | 28 |  | 1 |  | (0.6%) |
| interferon pegyle alfa 2b/ribavirin |  | 24 |  | 2 |  | (1.2%) |
| interferon pegyle alfa 2b/ribavirin |  | 48 |  | 1 |  | (0.6%) |
| interferon pegyle alfa 2a/ribavirin + sofosbuvir |  | 12 |  | 4 |  | (2.3%) |
| interferon pegyle alfa 2a/ribavirin + telaprevir |  | 24^b^ |  | 1 |  | (0.6%) |
| interferon pegyle alfa 2a/ribavirin + boceprevir |  | 24 |  | 1 |  | (0.6%) |
| interferon pegyle alfa 2a/ribavirin + telaprevir |  | 48^c^ |  | 5 |  | (2.9%) |
| interferon pegyle alfa 2a/ribavirin + boceprevir |  | 48^d^ |  | 1 |  | (0.6%) |
| interferon pegyle alfa 2a/ribavirin + sofosbuvir + ribavirin |  | 24^e^ |  | 1 |  | (0.6%) |
| interferon pegyle alfa 2b/ribavirin /boceprevir |  | 28 |  | 3 |  | (1.8%) |
| interferon pegyle alfa 2b/ribavirin /boceprevir |  | 48 |  | 1 |  | (0.6%) |
|  |  |  |  |  |  |  |

^a^ ledispavir/sofosbuvir (12 weeks) + ribavirin (24 weeks) + sofosbuvir (12 weeks, 12 weeks after initiating the other treatments)

^b^ peginterferon alfa-2a/ribavirin (24 weeks) + telaprevir (12 weeks)

^c^ peginterferon alfa-2a/ribavirin (48 weeks) + telaprevir (12 weeks)

^d^ peginterferon alfa-2a/ribavirin (48 weeks) + boceprevir (36 weeks)

^e^ Peginterferon alfa-2a/ribavirin (12 weeks) + sofosbuvir (24 weeks) + ribavirin (12 weeks, 12 weeks after initiating the other treatments)
