# Supplementary material for: Adherence and sustained virologic response among vulnerable people initiating an hepatitis C treatment at a nurse-led clinic: A non-experimental prospective cohort study based on clinical records
Source: Int J Nurs Stud Adv. 2021 May 26;3:100029. doi: 10.1016/j.ijnsa.2021.100029 (PMC11080331; doi:10.1016/j.ijnsa.2021.100029)
Supplement: Supplementary file 5 [file mmc5.docx]

## Additional file 6

### e-Table 2. Patients’ Characteristics Associated With Adherence to Hepatis C Treatment: Univariate Regression Analyses

| **Characteristics** | **N** |  | **Adherence to hepatitis C treatment** | |  | **P-value ^a^** |
| --- | --- | --- | --- | --- | --- | --- |
|  |  |  | **N** | **(%)** |  |  |
| **Sociodemographic characteristics** |  |  |  |  |  |  |
| Sex |  |  |  |  |  | 0.1820 |
| Male | 114 |  | 94 | (82.5%) |  |  |
| Female | 44 |  | 32 | (72.7%) |  |  |
| Sexual orientation |  |  |  |  |  | 0.8196 |
| Heterosexual | 152 |  | 121 | (79.6%) |  |  |
| Other | 6 |  | 5 | (83.3%) |  |  |
| Age (years) |  |  |  |  |  | **0.0326** |
| 22 to 45: quantile 1 | 40 |  | 27 | (67.5%) |  |  |
| 46 to 52: quantile 2 | 43 |  | 33 | (76.7%) |  |  |
| 53 to 59: quantile 3 | 35 |  | 29 | (82.9%) |  |  |
| 60 to 75: quantile 4 | 40 |  | 37 | (92.5%) |  |  |
| Country of birth |  |  |  |  |  | 0.5544 |
| Canada | 150 |  | 119 | (79.3%) |  |  |
| Other | 8 |  | 7 | (87.5%) |  |  |
| Marital status |  |  |  |  |  | 0.2873 |
| Single | 101 |  | 78 | (77.2%) |  |  |
| In relationship | 57 |  | 48 | (84.2%) |  |  |
| Housing |  |  |  |  |  | 0.5619 |
| Home | 147 |  | 118 | (80.3%) |  |  |
| Homeless | 11 |  | 8 | (72.7%) |  |  |
| Place of residence |  |  |  |  |  | 0.3552 |
| [PLACE HIDDEN], [PLACE HIDDEN] district | 85 |  | 66 | (77.6%) |  |  |
| [PLACE HIDDEN], any other district | 37 |  | 32 | (86.5%) |  |  |
| Outside [PLACE HIDDEN], but within the [PLACE HIDDEN] administrative region | 11 |  | 10 | (90.9%) |  |  |
| Outside the [PLACE HIDDEN] administrative region | 25 |  | 18 | (72.0%) |  |  |
| College or university degree |  |  |  |  |  | 0.1415 |
| No | 111 |  | 85 | (76.6%) |  |  |
| Yes | 33 |  | 29 | (87.9%) |  |  |
| Missing data | 14 |  | 12 | (85.7%) |  |  |
| Primary occupation |  |  |  |  |  | 0.5282 |
| Work, study, or retired | 72 |  | 59 | (81.9%) |  |  |
| Unemployed | 86 |  | 67 | (77.9%) |  |  |
| Monthly income |  |  |  |  |  | 0.2662 |
| From US$375 to $749 | 85 |  | 65 | (76.5%) |  |  |
| ≥ US$750 | 73 |  | 61 | (93.6%) |  |  |
| Medication insurance coverage |  |  |  |  |  | 0.8891 |
| Public | 127 |  | 101 | (79.5%) |  |  |
| Private | 31 |  | 25 | (80.6%) |  |  |
| Have a criminal record |  |  |  |  |  | 0.0882 |
| No | 65 |  | 56 | (86.2%) |  |  |
| Yes | 93 |  | 70 | (75.3%) |  |  |
| **Health** |  |  |  |  |  |  |
| Body mass index (weight/height^2^) |  |  |  |  |  | 0.4670^c^ |
| <24.9 kg/m^2^: underweight or normal weight | 54 |  | 40 | (74.1%) |  |  |
| 25 to29.9 kg/m^2^: overweight | 55 |  | 46 | (83.6%) |  |  |
| >30 kg/m^2^: Obese | 30 |  | 24 | (80.0%) |  |  |
| Missing data | 19 |  | 16 | (84.2%) |  |  |
| Comorbid physical health problem |  |  |  |  |  | 0.2286 |
| No | 50 |  | 37 | (74.0%) |  |  |
| Yes | 108 |  | 89 | (82.4%) |  |  |
| Comorbid mental health problem |  |  |  |  |  | **0.0116** |
| No | 33 |  | 31 | (93.9%) |  |  |
| Yes | 125 |  | 95 | (76.0%) |  |  |
| **Behavior** |  |  |  |  |  |  |
| Self-reported drug use in the past 30 days prior to treatment initiation |  |  |  |  |  | **0.0126** |
| No | 75 |  | 66 | (88.0%) |  |  |
| Yes | 83 |  | 60 | (72.3%) |  |  |
| Self-reported alcohol consumption in the past 30 days prior to treatment initiation ^b^ |  |  |  |  |  | 0.0790 |
| Low-risk drinking | 125 |  | 104 | (83.2%) |  |  |
| High-risk drinking | 32 |  | 22 | (68.8%) |  |  |
| Currently smoking |  |  |  |  |  | 0.1578 |
| No | 56 |  | 48 | (85.7%) |  |  |
| Yes | 102 |  | 78 | (76.5%) |  |  |
| **Hepatitis C virus** |  |  |  |  |  |  |
| Hepatitis C virus genotype |  |  |  |  |  | 0.2529 |
| 1a | 86 |  | 69 | (80.2%) |  |  |
| 1b | 14 |  | 13 | (92.9%) |  |  |
| 2 | 12 |  | 11 | (91.7%) |  |  |
| 3 | 40 |  | 28 | (70.0%) |  |  |
| 4, 5, 6, and 1+3 | 6 |  | 5 | (83.3%) |  |  |
| Hepatitis C virus infection |  |  |  |  |  | 0.6501 |
| Exposure to blood infected with HCV through injection drug use | 129 |  | 102 | (79.1%) |  |  |
| Other mode of transmission ^c^ | 29 |  | 24 | (82.8%) |  |  |
| Liver fibrosis |  |  |  |  |  | 0.7591^c^ |
| Absent/mild, moderate, or severe fibrosis (F3-F0) | 67 |  | 52 | 77.6 |  |  |
| Advanced fibrosis (F4) or cirrhosis | 74 |  | 59 | 79.7 |  |  |
| Missing data | 17 |  | 15 | 88.2 |  |  |
| **Hepatitis C treatment** |  |  |  |  |  |  |
| Any prior hepatitis C treatment |  |  |  |  |  | 0.2774 |
| No | 106 |  | 82 | (77.4%) |  |  |
| Yes | 52 |  | 44 | (84.6%) |  |  |
| Current regimen prescribed |  |  |  |  |  | **0.0301** |
| Direct-acting antiviral without Ribavirin | 100 |  | 84 | (84.0%) |  |  |
| Direct-acting antiviral with Ribavirin | 34 |  | 28 | (82.4%) |  |  |
| Pegylated Interferon + Ribavirin | 24 |  | 14 | (58.3%) |  |  |
| Length of treatment |  |  |  |  |  | **0.0010** |
| 8 or 12 weeks | 124 |  | 106 | (85.5%) |  |  |
| 24 or 28 weeks | 27 |  | 18 | (66.7%) |  |  |
| 48 weeks | 7 |  | 2 | (28.6%) |  |  |
| ≥1 self-reported adverse reaction |  |  |  |  |  | 0.1712 |
| No | 66 |  | 56 | (84.8%) |  |  |
| Yes | 92 |  | 70 | (76.1%) |  |  |
| **Medication and health service use** |  |  |  |  |  |  |
| Number of concomitant prescribed medications | 14 |  | 13 | (92.9%) |  | 0.2628 |
| 0 | 59 |  | 43 | (72.9%) |  |  |
| 1 to 4 | 53 |  | 43 | (81.1%) |  |  |
| 5 to 9 | 32 |  | 27 | (84.4%) |  |  |
| ≥10 |  |  |  |  |  |  |
| Family doctor | 99 |  | 82 | (82.8%) |  | 0.2164 |
| Yes | 59 |  | 44 | (74.6%) |  |  |
| No |  |  |  |  |  |  |

^a^ For these analyses, we built univariate logistic regression models. P values are derived from exact tests.

^b^ In women, low-risk drinking is defined as no more than 10 standard drinks a week and as no more than 3 drinks a day or 15 drinks a week in men. One standard drink is equivalent to one regular beer (340 ml/12 oz, 5% alcohol), one glass of wine (140 ml/5oz, 12% alcohol), one glass of fortified wine (85 ml/3oz, 20% alcohol) and one shot of spirits (45 ml/1.5 oz, 40% alcohol).

^c^ Other modes of transmission include: using intranasal drugs; recipients of blood transfusions; being born to a mother who are infected with hepatitis C virus; unregulated tattooing and piercing in prisons, etc.
