# Supplementary material for: Adherence and sustained virologic response among vulnerable people initiating an hepatitis C treatment at a nurse-led clinic: A non-experimental prospective cohort study based on clinical records
Source: Int J Nurs Stud Adv. 2021 May 26;3:100029. doi: 10.1016/j.ijnsa.2021.100029 (PMC11080331; doi:10.1016/j.ijnsa.2021.100029)
Supplement: Supplementary file 6 [file mmc6.docx]

## Additional File 7

### e-Table 3. Patients’ Characteristics Associated with Sustained Virologic Response: Univariate Regression Analyses

| **Characteristics** | **N** |  | **Sustained virologic response** | |  | **P-value ^a^** |
| --- | --- | --- | --- | --- | --- | --- |
|  |  |  | **N** | **(%)** |  |  |
|  |  |  |  |  |  |  |
| **Sociodemographic characteristics** |  |  |  |  |  |  |
| Sex |  |  |  |  |  | 0.8276 |
| Male | 120 |  | 105 | (87.5%) |  |  |
| Female | 51 |  | 44 | (86.3%) |  |  |
| Sexual orientation |  |  |  |  |  | 0.3881 |
| Heterosexual | 165 |  | 143 | (86.7%) |  |  |
| Other | 6 |  | 6 | (100%) |  |  |
| Age (years) |  |  |  |  |  | 0.0589 |
| 22 to 45: quantile 1 | 45 |  | 36 | (80.0%) |  |  |
| 46 to 52: quantile 2 | 47 |  | 40 | (85.1%) |  |  |
| 53 to 59: quantile 3 | 39 |  | 34 | (87.2%) |  |  |
| 60 to 75: quantile 4 | 40 |  | 39 | (97.5%) |  |  |
| Country of birth |  |  |  |  |  | 0.2332 |
| Canada | 162 |  | 140 | (86.4%) |  |  |
| Elsewhere | 9 |  | 9 | (100%) |  |  |
| Marital status |  |  |  |  |  | 0.1445 |
| Single | 109 |  | 92 | (84.4%) |  |  |
| In relationship | 62 |  | 57 | (91.9%) |  |  |
| Housing |  |  |  |  |  | 0.6078 |
| Home | 159 |  | 138 | (86.8%) |  |  |
| Homeless | 12 |  | 11 | (91.7%) |  |  |
| College or university degree |  |  |  |  |  | **0.0113** |
| No | 120 |  | 101 | (84.2%) |  |  |
| Yes | 34 |  | 34 | (100%) |  |  |
| Missing data | 17 |  | 14 | (82.4%) |  |  |
| Primary occupation |  |  |  |  |  | **0.0276** |
| Work, study, or retired | 74 |  | 70 | (94.6%) |  |  |
| Unemployed | 97 |  | 79 | (81.4%) |  |  |
| Monthly income |  |  |  |  |  | **0.0100** |
| From US$375 to $749 | 95 |  | 78 | (82.1%) |  |  |
| ≥ US$750 | 75 |  | 71 | (94.7%) |  |  |
| Medication insurance coverage |  |  |  |  |  | **0.0373** |
| Public | 139 |  | 118 | (84.9%) |  |  |
| Private | 32 |  | 31 | (96.9%) |  |  |
| Have a criminal record |  |  |  |  |  | **0.0035** |
| No | 69 |  | 66 | (95.7%) |  |  |
| Yes | 102 |  | 83 | (81.4%) |  |  |
|  |  |  |  |  |  |  |
| **Health** |  |  |  |  |  |  |
| Body mass index (weight/height^2^) |  |  |  |  |  | 0.2975^c^ |
| <24.9 kg/m^2^: underweight or normal weight | 59 |  | 54 | 91.5 |  |  |
| 25 to29.9 kg/m^2^: overweight | 58 |  | 53 | 91.4 |  |  |
| >30 kg/m^2^: Obese | 32 |  | 26 | 81.3 |  |  |
| Missing data | 22 |  | 16 | 72.7 |  |  |
| Comorbid physical health problem |  |  |  |  |  | 0.9205 |
| No | 56 |  | 49 | (87.5%) |  |  |
| Yes | 115 |  | 100 | (87.0%) |  |  |
| Comorbid mental health problem |  |  |  |  |  | **0.0198** |
| No | 36 |  | 35 | (97.2%) |  |  |
| Yes | 135 |  | 114 | (84.4%) |  |  |
|  |  |  |  |  |  |  |
| **Behavior** |  |  |  |  |  |  |
| Self-reported illicit drug use in the 30 days prior to HCV treatment initiation |  |  |  |  |  | 0.0787 |
| No | 84 |  | 77 | (91.7%) |  |  |
| Yes | 87 |  | 72 | (82.8%) |  |  |
| Self-reported alcohol consumption in the 30 days prior to HCV treatment initiation ^b^ |  |  |  |  |  | 0.2388 |
| Low-risk drinking | 133 |  | 118 | 88.7 |  |  |
| High-risk drinking | 37 |  | 30 | 81.1 |  |  |
| Currently smoking |  |  |  |  |  | **0.0184** |
| No | 59 |  | 56 | (94.9%) |  |  |
| Yes | 112 |  | 93 | (83.0%) |  |  |
|  |  |  |  |  |  |  |
| **Hepatitis C Virus** |  |  |  |  |  |  |
| Hepatitis C virus genotype |  |  |  |  |  | 0.3338 |
| 1a | 93 |  | 82 | (88.2%) |  |  |
| 1b | 15 |  | 14 | (93.3%) |  |  |
| 2 | 13 |  | 12 | (92.3%) |  |  |
| 3 | 43 |  | 34 | (79.1%) |  |  |
| 4, 5, 6, and 1+3 | 7 |  | 7 | (100%) |  |  |
| Hepatitis C virus infection |  |  |  |  |  | **0.0436** |
| Exposure to blood infected with HCV through injection drug use | 140 |  | 119 | (85.0%) |  |  |
| Other mode of transmission ^c^ | 31 |  | 30 | (96.8%) |  |  |
| Liver fibrosis |  |  |  |  |  | 0.8909^c^ |
| Absent/mild, moderate, or severe fibrosis (F3-F0) | 74 |  | 64 | (86.5%) |  |  |
| Advanced fibrosis (F4) or cirrhosis | 77 |  | 66 | (85.7%) |  |  |
| Missing data | 20 |  | 19 | (95.0%) |  |  |
|  |  |  |  |  |  |  |
| **Hepatitis C treatment** |  |  |  |  |  |  |
| Any prior Hepatitis C treatment |  |  |  |  |  |  |
| No | 115 |  | 98 | (85.2%) |  | 0.2700 |
| Yes | 56 |  | 51 | (91.1%) |  |  |
| Current regimen prescribed |  |  |  |  |  | 0.6887 |
| Direct-acting antiviral without Ribavirin | 106 |  | 93 | (87.7%) |  |  |
| Direct-acting antiviral with Ribavirin | 37 |  | 33 | (89.2%) |  |  |
| Pegylated Interferon + Ribavirin | 28 |  | 23 | (82.1%) |  |  |
| Length of treatment |  |  |  |  |  | 0.5787 |
| 8 or 12 weeks | 133 |  | 116 | (87.2%) |  |  |
| 24 or 28 weeks | 30 |  | 27 | (90.0%) |  |  |
| 48 weeks | 8 |  | 6 | (75.0%) |  |  |
| Adherent to HCV treatment |  |  |  |  |  | **0.0024** |
| Yes | 126 |  | 116 | (92.1%) |  |  |
| No | 45 |  | 33 | (73.3%) |  |  |
| ≥1 self-reported adverse reaction |  |  |  |  |  | 0.7134^c^ |
| No | 66 |  | 60 | (90.6%) |  |  |
| Yes | 92 |  | 82 | (89.1%) |  |  |
| Missing data | 13 |  | 7 | (53.8%) |  |  |
|  |  |  |  |  |  |  |
| **Medication and healthcare service use** |  |  |  |  |  |  |
| Number of concomitant prescribed medications |  |  |  |  |  | 0.5013 |
| 0 | 16 |  | 15 | (93.8%) |  |  |
| 1 to 4 | 65 |  | 57 | (87.7%) |  |  |
| 5 to 9 | 55 |  | 49 | (89.1%) |  |  |
| ≥10 | 35 |  | 28 | (80.0%) |  |  |
| Family doctor |  |  |  |  |  | 0.9911 |
| Yes | 109 |  | 95 | (87.2%) |  |  |
| No | 62 |  | 54 | (87.1%) |  |  |
|  |  |  |  |  |  |  |

^a^ For these analyses, we built univariate logistic regression models. P-values are derived from exact tests.

^b^ In women, low-risk drinking is defined as no more than 10 standard drinks a week and as no more than 3 drinks a day or 15 drinks a week in men. One standard drink is equivalent to one regular beer (340 ml/12 oz, 5% alcohol), one glass of wine (140 ml/5oz, 12% alcohol), one glass of fortified wine (85 ml/3oz, 20% alcohol) and one shot of spirits (45 ml/1.5 oz, 40% alcohol).

^c^ Other modes of transmission include: using intranasal drugs; recipients of blood transfusions; being born to a mother who are infected with hepatitis C virus; unregulated tattooing and piercing in prisons, etc.
